# Effect of high glucose supplementation on pulmonary fibrosis involving reactive oxygen species and TGF-β

**DOI:** 10.3389/fnut.2022.998662

**Published:** 2022-10-11

**Authors:** Wenjuan Ning, Xiaoxiao Xu, Shican Zhou, Xiao Wu, Hang Wu, Yijie Zhang, Jichang Han, Junpeng Wang

**Affiliations:** Infection and Immunity Institute and Translational Medical Center of Huaihe Hospital, Henan University, Kaifeng, China

**Keywords:** diet, glucose, ROS, latent TGF-β1, epithelial–mesenchymal transition, pulmonary fibrosis

## Abstract

This study explored the profibrotic impact of high glucose in the lung and potential mechanisms using latent TGF-β1-induced human epithelial cell pulmonary fibrosis and bleomycin (BLM)-induced pulmonary fibrosis models. Results demonstrated that high glucose administration induced epithelial–mesenchymal transition (EMT) in human epithelial cells in a dose-dependent manner *via* activating latent TGF-β1, followed by increased expression of mesenchymal-related proteins and decreased expression of epithelial marker protein E-cadherin. Further mechanism analysis showed that administration of high glucose dose-dependently promoted total and mitochondrial reactive oxygen species (ROS) accumulation in human epithelial cells, which promoted latent TGF-β1 activation. However, *N*-acetyl-L-cysteine, a ROS eliminator, inhibited such effects. An *in vivo* feed study found that mice given a high-glucose diet had more seriously pathological characteristics of pulmonary fibrosis in BLM-treated mice, including increasing infiltrated inflammatory cells, collagen I deposition, and the expression of mesenchymal-related proteins while decreasing the expression of the epithelial marker E-cadherin. In addition, high glucose intake further increased TGF-β1 concentration and upregulated p-Smad2/3 and snail in lung tissues from BLM-treated mice when compared to BLM-treated mice. Finally, supplementation with high glucose further increased the production of lipid peroxidation metabolite malondialdehyde and decreased superoxide dismutase activity in BLM-treated mice. Collectively, these findings illustrate that high glucose supplementation activates a form of latent TGF-β1 by promoting ROS accumulation and ultimately exacerbates the development of pulmonary fibrosis.

## Introduction

The most common interstitial lung disease is idiopathic pulmonary fibrosis (IPF), which has an unknown etiology and poor prognosis (1). IPF is primarily characterized by the continuous damage of alveolar epithelial cells, resulting in fibroblast activation, differentiation, and extracellular matrix (ECM) accumulation. It gradually causes structural damage and a decline in lung function (2). In IPF patients, the median survival time was only 2.5∼3.5 years (3), and morbidity was 1.7–27.1 per year for every 100,000 people (4, 5).

The epithelial–mesenchymal transition (EMT) refers to the changes in cell phenotype as epithelial cells transition to mesenchymal cells. EMT is indispensable in the development, wound healing, and stem cell behavior (6) and promotes pathologically fibrosis in the kidney, liver, intestine, and lung (7–9). Evidence shows that many signaling pathways are required for EMT. During the EMT process, extracellular signals can initiate and regulate gene expression reprogramming. Especially, TGF-β family signals play a critical role in EMT (10).

TGF-β, the most important profibrotic growth factor, regulates gene expression, cell survival, proliferation, differentiation, and apoptosis (11). TGF-β is abundant in fibrotic lung tissues and can be secreted by various cells, including neutrophils, alveolar epithelial cells, alveolar macrophages, and fibroblasts (12). TGF-β usually exists in a latent form in the ECM and can only function when activated *via* αv integrins, reactive oxygen species (ROS), plasmin, and matrix metalloproteinases (13–15). Currently, the primary target of pulmonary fibrosis drug treatment is to inhibit the effect of TGF-β, but this does not reverse lung fibrosis or provide additional survival benefits (16, 17).

Dietary nutrients have been identified as a potential environmental risk factor for the rise in chronic diseases such as metabolic syndrome, cardiovascular diseases, and autoimmune diseases in humans. High salt intake, for example, exacerbated the development of experimental autoimmune encephalomyelitis (EAE), an animal model of multiple sclerosis, by promoting Th17 cell differentiation (18). A high carbohydrate diet may be a detrimental risk factor for cardiovascular diseases, whereas unsaturated fatty acids may reduce these risk factors (19); and feeding rhesus with free-cholesterol low-fat and high carbohydrates for 7 years demonstrated for the first time that a high fructose diet causes liver fat and liver fibrosis in rhesus monkeys (20).

Glucose, an intermediate product of cellular energy source and metabolism, is the primary energy supply substance of the human body. Adults get 55–60% of their daily energy from carbohydrates. A high-glucose diet is closely linked to human health, particularly for the Asian populations who consume grains and other carbohydrates. Thus, paying attention to changes in the structure of high glucose diet is related to health. High glucose supplementation may increase ROS production, altering inflammatory mediators and neurodegenerative markers in retina RGC-5 cells (21), as well as activate latent TGF-β1 to promote Th17 cell differentiation, worsening autoimmunity, including inflammatory bowel disease (an animal model of ulcerative colitis) and EAE (22). Notably, Wang et al. have proposed that persistent hyperglycemia type 2 diabetes may contribute to IPF development (23). However, whether high glucose intake affects pulmonary fibrosis progression in a short-term feeding experiment needs to be investigated.

Thus, in this study, we aimed to investigate the effects of excessive glucose intake on pulmonary fibrosis utilizing the EMT process of TGF-β1-induced A549 cells *in vitro* and bleomycin (BLM)-induced pulmonary fibrosis in mice and the underlying mechanisms.

## Materials and methods

### Materials

α-D-Glucose (Glu, #G8150) was purchased from Beijing Solarbio. Shanghai Aladdin and MCE provided N-Acetyl-l-cysteine (NAC, #A105422) and SB431542 (HY-10431), respectively. Recombinant human latent TGF-β1 (L-TGF-β, #299-LT) and recombinant human TGF-β1 (#240-B) were purchased from R&D Systems. Bleomycin hydrochloride was from Hanhui Pharmaceutical Company (Hangzhou, China). Nitrocellulose membranes (#66485) were obtained from Pall. DCFH-DA (#MX4802) and MitoSOX™ Red mitochondrial superoxide indicator (#M36008) were purchased from Shanghai Maokang and ThermoFisher, respectively.

### Cell culture

Human type II alveolar epithelial cells (A549) were cultured in DMEM containing 5% fetal bovine serum and 1% penicillin/streptomycin in an incubator at 37°C with 5% CO_2_. Cells were administrated, as indicated, with different doses of glucose in the presence or absence of L-TGF-β (10 ng/ml), TGF-β1 (5 ng/ml), SB431542 (5 μM), or NAC (5 mM). A microscope was used to recognize cell morphological alterations in A549 cells.

### Cell viability

We seeded A549 cells in a 96-well plate and then cultured them with glucose under the indicated dose for 24 h; a CCK-8 solution was added for another 1 h to determine cell viability using a microplate reader (SpectraMax i3x) at 450 nm. The cell viability was calculated using this formula: (absorbance of glucose-treated sample/absorbance of control sample) × 100.

### Detection of reactive oxygen species

A549 cells were cultured for 24 h with various concentrations of glucose (0, 10, 20, and 50 mM) and then were treated with DCFH-DA at 10 μM for another 20 min to determine the total intracellular ROS or MitoSox™ at 2.5 μM for another 10 min to determine the mitochondrial ROS. The fluorescence of cells was then measured using fluorescence microscopy (OLYMPUS, Tokyo, Japan), a microplate reader (SpectraMax i3x), or flow cytometry (BD FACSCanto Plus). Flowjo 10.0.7 software was used to analyze flow cytometry data.

### Animals

Male C57BL/6 mice (6–8 weeks) were purchased from the Nanjing Biomedical Research Institute of Nanjing University (Nanjing, China). A nutritionally adequate standard diet formulated for rodents were purchased from Beijing Keao Xieli Feed Co., Ltd. (Beijing, China). This commercial diet contains 19.53% crude protein, 4.2% fat, 4.7% crude fiber, 6.2% crude ash, 1.07% calcium, and 0.81% phosphorus. This diet was adequate in all nutrients and met the rodent’s nutrition requirements. The mice were divided into four groups at random: Control group, BLM group, BLM + Glu group, and Glu group. After 2 weeks of regular water with or without containing 20% glucose, mice were anesthetized intramuscularly with a ketamine (60 mg/kg)/xylazine (1.25 mg/kg) solution and then instilled intratracheally with BLM (2.5 mg/kg) or saline. Mice were fed the same water after instillation until the end of this study. All experimental procedures were carried out following the Guide for the Laboratory Animals by the National Institute of Health and were approved by the Institutional Animal Care and Use Committee of Huaihe Hospital at Henan University.

### Lung histopathology

Mice were euthanized with CO_2_, and the left lobe was collected and then fixed with 10% formalin for the histopathological analysis on day 21 after BLM instillation. Hematoxylin–eosin (H&E) and Masson’s staining were used to assess inflammatory cell infiltration and pulmonary interstitial fibrosis. The Szapiel score was used to evaluate alveolar inflammation (24), and the Ashcroft score was used to evaluate pulmonary fibrosis (25).

### Enzyme-linked immunosorbent assay

After mice were euthanized with CO_2_ on day 21 following BLM instillation, plasma and the right lung were collected. Lung homogenates were prepared as previously described (26). TGF-β1 enzyme-linked immunosorbent assay (ELISA) kit (eBioscience, San Diego) was used to detect the level of TGF-β1 in mouse lung homogenates and plasma.

### Measurement of superoxide dismutase and malondialdehyde

On day 21 after BLM instillation, the right lung was collected, and lung homogenates were prepared as described previously (26) to determine malondialdehyde (MDA) levels and superoxide dismutase (SOD) activity using the MDA and SOD kit from Nanjing JianCheng Bioengineering Institute according to the instructions.

### Western blot

Total protein was extracted from lung or A549 cells and homogenized in RIPA lysis buffer with protease inhibitor and phosphatase inhibitor cocktails before being quantified using a BCA protein assay kit (Shanghai EpiZyme Biotechnology Co., Ltd.). Total protein extraction was resolved by 10% acrylamide gel before being transferred to the nitrocellulose membrane. The nitrocellulose membrane was blocked for 1 h at room temperature with 5% non-fat milk. They were then incubated overnight at 4°C with the following specific primary antibodies: N-Cadherin (1:1,000), E-Cadherin (1:1,000), Vimentin (1:1,000), p-Smad2/3 (1:1,000), Snail (1:1,000) (all from Cell Signaling Technologies, Danvers, MA), α-Smooth muscle actin (α-SMA) (1:1,000, SinoBiological Inc., Beijing, China), Collagen I (1:1,000, Bioss, Beijing, China), and β-actin (1:5,000, Sigma-Aldrich). Finally, the membranes were incubated for 1 h at room temperature with horseradish peroxidase-conjugated anti-mouse or anti-rabbit secondary antibodies before exposing them to enhanced chemiluminescent reagents (Solarbio^®^ Life Science, Beijing, China).

### Statistical analysis

The results were presented as mean ± SEM. GraphPad Prism 8.0 was used for statistical analyses. A one-way analysis of variance was used to determine differences, followed by Tukey’s HSD *post-hoc* test. *P* < 0.05 indicated that the findings were statistically significant.

## Results

### Effect of high glucose on A549 cell viability

**FIGURE 1:**
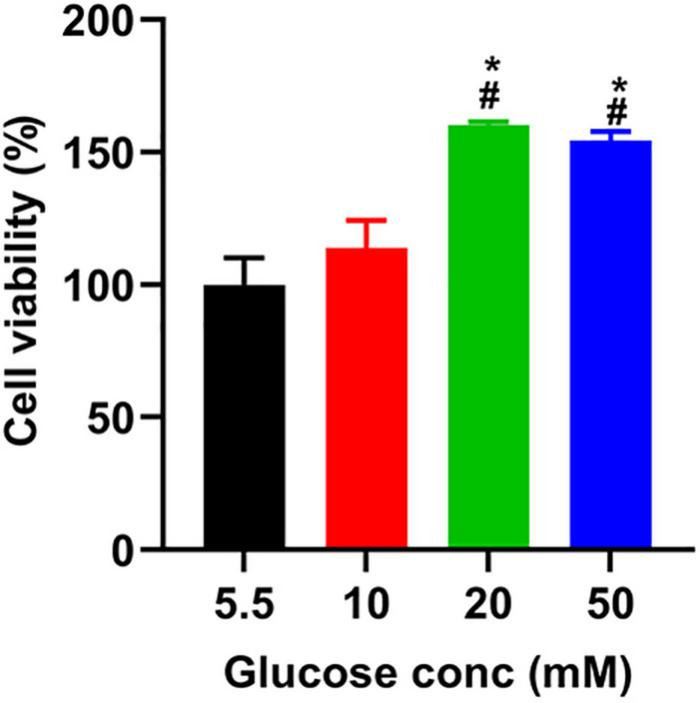
The effect of glucose on the viability of A549 cells. After incubating A549 cells with glucose at the indicated concentrations for 24 h, we added a CCK-8 solution to each well and incubated them for another 1 h. The absorbance was measured at 450 nm, described in the section “Materials and Methods.” *P < 0.05 vs. 5.5 mM glucose; ^#^*P* < 0.05 vs. 10 mM glucose. Values are the means ± SEM (*n* = 3).

To determine the toxicity of glucose on A549 cells, we used a CCK8 assay to assess the viability of A549 cells treated with different doses of glucose for 24 h. Compared to the control group (5.5 mM), glucose dose-dependently promoted A549 cell viability (Figure 1).

### High glucose promotes latent TGF-β1-induced morphological alterations in A549 cells

**FIGURE 2:**
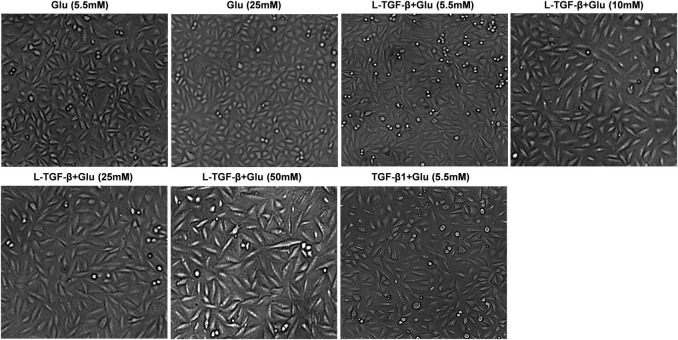
High glucose promoted latent TGF-β1-induced morphological alteration in A549 cells. Cell morphological images were taken under a microscope after A549 cells were co-cultured in DMEM medium with glucose (5.5, 10, 25, and 50 mM) with/without human latent TGF-β1 (10 ng/mL) for 24 h. Representative images per group are shown (*n* = 3). L-TGF-β, latent TGF-β1; Glu, glucose.

We co-cultured A549 cells with the indicated glucose concentration in the presence or absence of latent TGF-β1 for 24 h to see if high glucose impacts the EMT process of A549 cells treated by latent TGF-β1. As shown in Figure 2, latent TGF-β1 or glucose (20 mM) alone did not induce the EMT in A549 cells compared to the control group (5.5 mM). However, latent TGF-β1 significantly altered cell morphology when combined with high glucose administration (10, 20, and 50 mM). These findings suggested that high glucose may cause morphological alterations in latent TGF-β1-treated A549 cells.

### High glucose induces reactive oxygen species increase in A549 cells

**FIGURE 3:**
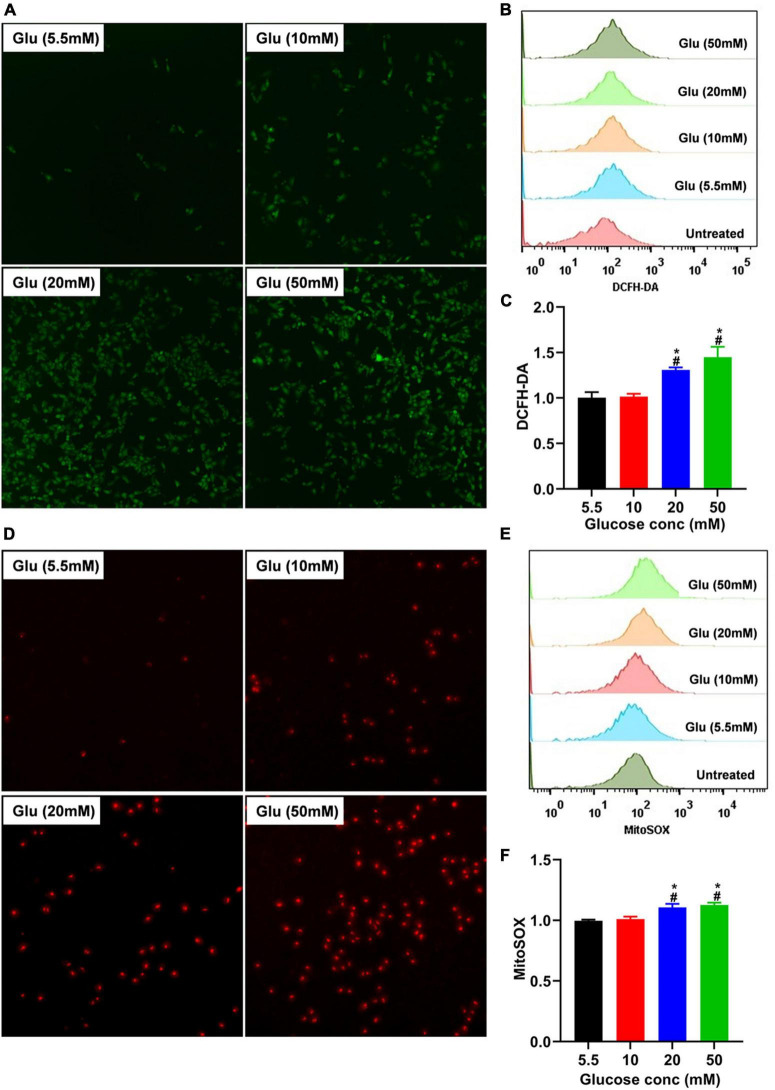
Effect of high glucose on the production of ROS in A549 cells. A549 cells were cultured in DMEM with glucose at 5.5, 10, 20, and 50 mM for 24 h and were stained with the DCFH-DA or MitoSox probe to determine total intracellular or mitochondrial ROS, respectively. The production of total **(A)** or mitochondrial **(D)** ROS was demonstrated by a representative fluorescent image for DCFH-DA or MitoSox. Flow cytometry was also used to determine the fluorescence of total and mitochondrial ROS, and the data were analyzed using the Flowjo software. **(B,E)** Show a representative histogram image for total or mitochondrial ROS. The summary data (*n* = 3) are shown in **(C,F)**. *P < 0.05 vs. control group; ^#^*P* < 0.05 vs. 10 mM glucose group. Glu, glucose.

TGF-β usually exists in a latent form and can only function when activated by several mechanisms, including ROS (27). To explore whether glucose’s effect on A549 cell morphology is mediated by ROS-induced latent TGF-β1 activation, we co-cultured A549 cells treated with different doses of glucose for 24 h and then used the DCFH-DA indicator and MitoSOX™ Red mitochondrial superoxide indicator to detect the total and mitochondrial ROS changes, respectively. As shown in Figure 3, glucose dose-dependently increased intracellular total and mitochondrial ROS production.

### High glucose alters the expression of latent TGF-β1-induced epithelial–mesenchymal transition-related proteins in A549 cells

**FIGURE 4:**
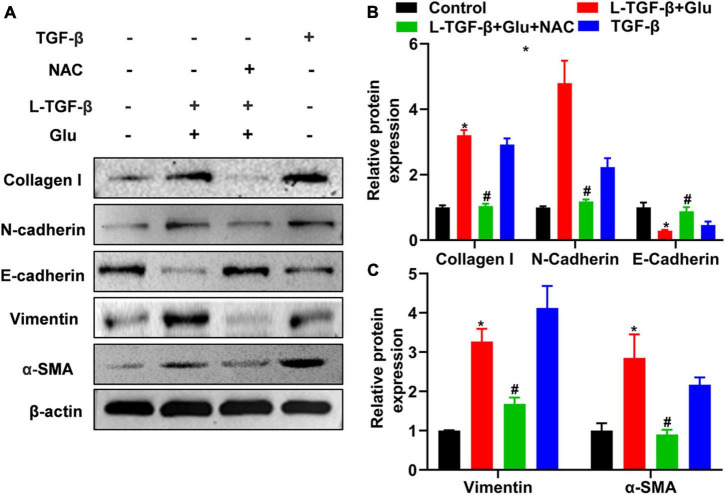
Glucose promotes latent TGF-β1 induced epithelial-mesenchymal transition of A549 cells. A549 cells were cultured for 24 h in 25 mM glucose, with or without human latent TGF-β1 (10 ng/mL), TGF-β1 (5 ng/ml), and NAC (5 mM). We used western blot to analyze the expression of Collagen I, N-Cadherin, E-cadherin, vimentin, and α-SMA in A549 cells. Representative gels were shown in **(A)** and the summary data (n = 3) were shown in **(B,C)**. *P < 0.05 vs. control group; ^#^*P* < 0.05 vs. Latent TGF-β1 + Glu (25 mM) group. L-TGF-β, latent TGF-β1, NAC, *N*-acetyl-L-cysteine; α1-SMA, α1-smooth muscle actin; Glu, glucose; TGF-β, TGF-β1.

To investigate whether glucose affects the EMT process of latent TGF-β1-treated A549 cells, we measured the EMT-related protein expression for 24 h after A549 cells co-cultured with latent TGF-β1 with/without glucose at 25 mM or TGF-β1 alone. In comparison to the control group (5.5 mM), consistent with TGF-β1-induced expression of EMT-related markers, co-administration of latent TGF-β1 with glucose (25 mM) increased the expression of N-cadherin, collagen I, α-SMA, and vimentin while decreased epithelial marker E-cadherin (Figure 4). Furthermore, we determined the expression of EMT-related markers in A549 cells treated with TGF-β1 with/without glucose at 25 mM, but we found no difference in the expression of EMT-related markers (data not shown).

**FIGURE 5:**
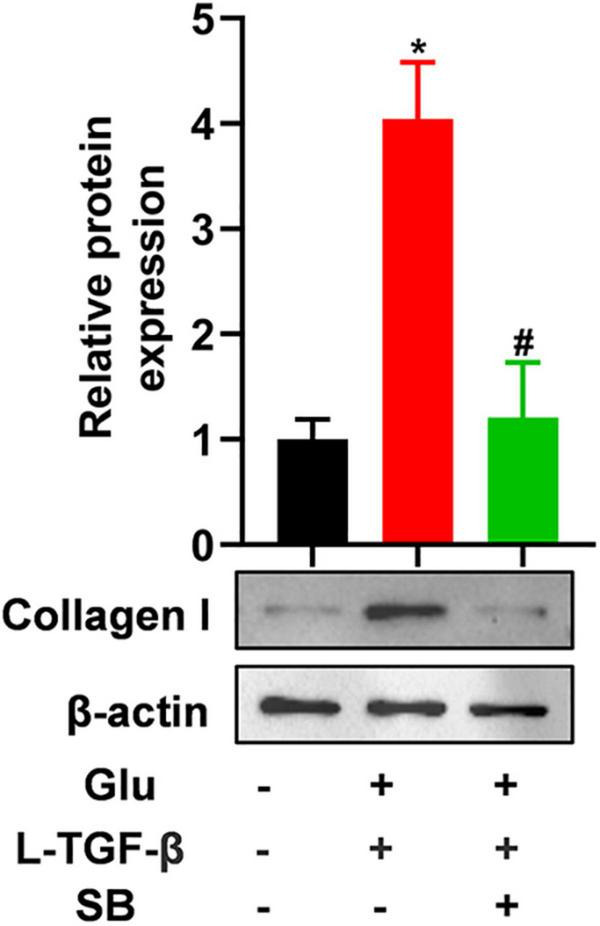
TGF-β receptor inhibitor blocks latent TGF-β1-induced EMT in glucose-treated A549 cells. We cultured A549 cells for 24 h with 25 mM glucose with or without human latent TGF-β1 (10 ng/mL), SB431542 (5 μM), and then performed a western blot assay to analyze the expression of Collagen I (*n* = 3). *P < 0.05 vs. control group; ^#^*P* < 0.05 vs. Latent TGF-β1 + Glu (25 mM) group. L-TGF-β, latent TGF-β1; SB, SB43152; Glu, glucose; TGF-β, TGF-β1.

To test whether latent TGF-β1 induces the EMT process in the presence of glucose, we blocked the TGF-β receptor using SB431542, a potent and selective inhibitor of TGF-β type I receptor, and discovered that the expression of mesenchymal marker collagen I was down-regulated in A549 cells treated with glucose and latent TGF-β1 (Figure 5). These findings confirmed that glucose activates the latent form of TGF-β1, which mediates the EMT process of A549 cells.

### *N*-acetyl-L-cysteine blocks epithelial–mesenchymal transition induced by latent TGF-β1 and glucose in A549 cells

We added NAC to A549 cells treated with glucose and latent TGF-β1 to explore whether latent TGF-β1 activation is caused by ROS production from glucose. As shown in Figure 4, administration with NAC reversed the expression of EMT-related markers compared to glucose combined with latent TGF-β1 or TGF-β1 alone, indicating that latent TGF-β1 activation may occur *via* glucose-derived ROS to induce the MET process in A549 cells.

### High glucose promotes latent TGF-β1-induced Smad2/3 activation and snail expression of A549 cells

**FIGURE 6:**
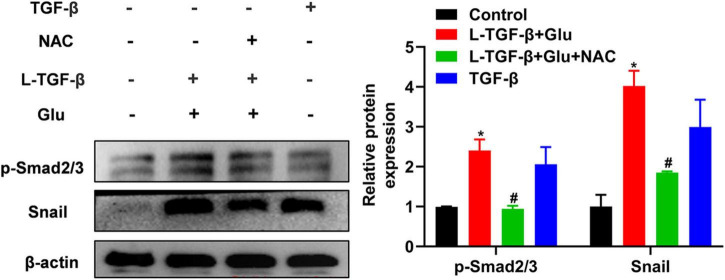
The effect of high glucose on the latent TGF-β1 induced Smad signal pathway in A549 cells. A549 cells were co-treated in the presence of 25 mM glucose with or without human latent TGF-β1 (10 ng/mL), TGF-β1 (5 ng/mL), and NAC (5 mM) for 24 h. The cells were collected and lysed for Western blot analysis (*n* = 3). Representative gels were shown in left panel and the summary data were shown in right panel. *P < 0.05 vs. control group; ^#^*P* < 0.05 vs. Latent TGF-β + Glu (25 mM) group. L-TGF-β, latent TGF-β1; NAC, *N*-acetyl-L-cysteine; Glu, glucose; TGF-β, TGF-β1.

To examine whether latent TGF-β1 activation by glucose-derived ROS affects TGF-β downstream signal Smad2/3 activation and EMT-related critical transcriptional factor Snail expression, we measured phosphorylated Smad2/3 and snail1 expression in A549 cells after 24 h of treatment with latent TGF-β1 and high glucose (25 mM) with/without NAC. Co-cultured latent TGF-β1 and glucose increased smad2/3 phosphorylation and Snail1 expression. However, NAC reversed these changes (Figure 6), indicating that high glucose activates the TGF-β/Smad signaling pathway by increasing ROS levels, resulting in EMT in A549 cells.

### A high-glucose diet exacerbates lung histopathological changes and pulmonary fibrosis in bleomycin-treated mice

**FIGURE 7:**
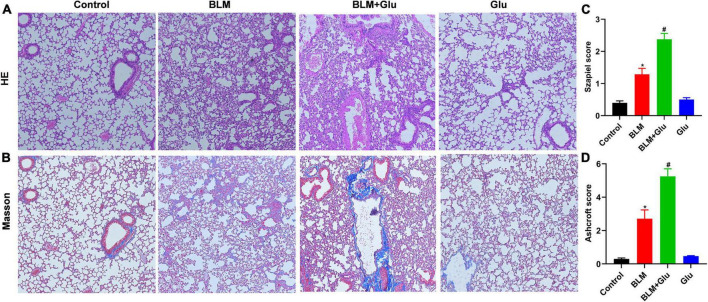
Pathological changes of pulmonary inflammation and fibrosis. **(A,B)** HE and Masson staining showed alveolar inflammation and pulmonary fibrosis (100 × magnification). **(C,D)** The Szapiel and Ashcroft scores were used to evaluate alveolar inflammation and pulmonary fibrosis, respectively. *P < 0.05 vs. control group; ^#^*P* < 0.05 vs. BLM group. The values are means ± SEM (*n* = 8/group). Glu, glucose; BLM, bleomycin.

**FIGURE 8:**
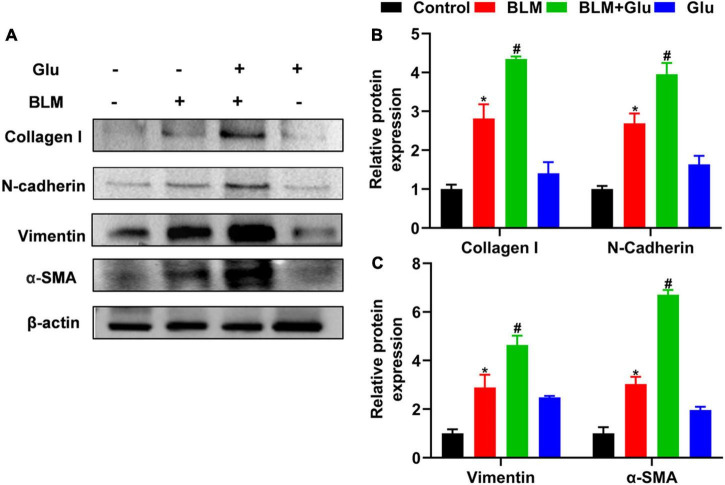
The effect of a high-glucose diet on BLM-induced pulmonary fibrosis in mice. We used western blot to analyze the expression of Collagen I, N-cadherin, Vimentin, and α-SMA. Representative gels were shown in **(A)** and the summary data were shown in **(B,C)**. *P < 0.05 vs. the control group; ^#^*P* < 0.05 vs. the BLM group. The values are means ± SEM (*n* = 3/group). Glu, 20% glucose; BLM, bleomycin.

The pathological characteristics of the control and Glu groups showed clear lung tissue structure and complete alveoli (Figure 7A). However, in the BLM-treated mice, the lung structure was destroyed, and alveolar inflammatory changes were observed; such was further aggravated in the mice treated with both glucose and BLM (Figures 7A,C). The analysis of Masson’s staining showed significant collagen deposition in lung tissues from BLM-treated mice when compared with control or glucose mice. However, compared to the BLM-treated mice, high dietary glucose further increased the lung collagen deposition in BLM-treated mice (Figures 7B,D), which was confirmed by an increase in collagen production (Figure 8). Finally, we found no differences in pulmonary histopathological alteration and fibrosis in mice supplemented with normal or high glucose water.

### A high glucose diet aggravates bleomycin-induced epithelial–mesenchymal transition-related protein expression

Compared to control mice, high glucose alone did not affect EMT-related protein expression, including N-cadherin, Vimentin, and α-SMA. However, BLM instillation increased these-related protein expressions, and this effect was amplified when mice were co-treated with high glucose and BLM (Figure 8).

### A high glucose diet increases lung TGF-β1 levels, Smad2/3 phosphorylation, and snail expression in mice

**FIGURE 9:**
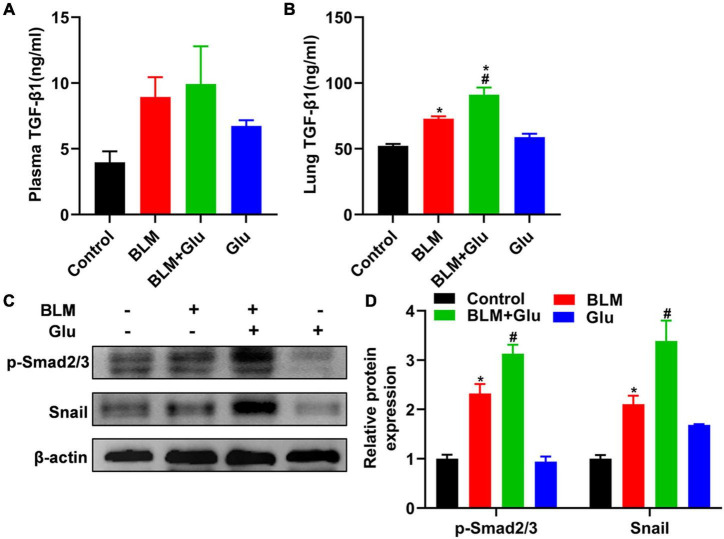
Effects of high-glucose diet on plasma and lung TGF-β1, Smad2/3 activation, and snail in mice. The levels of TGF-β1 in plasma **(A)** and lung homogenate **(B)** of mice were measured by ELISA. We used Western blot to analyze the expression of Smad2/3 and snail **(C,D)**. *P < 0.05 vs. the control group; ^#^*P* < 0.05 vs. BLM group. The values are means ± SEM (*n* = 8/group). Glu, glucose; BLM, bleomycin.

TGF-β has a decisive role in the induction of EMT in lung fibrosis. Therefore, we will determine whether the aggravating effect of high glucose on pulmonary fibrosis was caused by its modulation of TGF-β1 levels in BLM-treated mice. Compared to control mice, high glucose intake alone did not affect TGF-β1 levels in plasma or lung tissues (Figure 9A). Furthermore, BLM treatment increased lung TGF-β1 levels compared to mice supplemented with normal or high glucose alone (Figures 9A,B). However, high glucose did not increase plasma TGF-β1 but did increase lung TGF-β1 concentration in mice treated with BLM (Figures 9A,B).

Then, we looked at whether high glucose affects TGF-β downstream signal Smad2/3 activation and EMT transcriptional factor Snail1 expression. Mice supplemented with high glucose did not have a higher expression of Smad2/3 phosphorylation and Snail1 than that of mice fed with normal water. However, BLM treatment increased the expression of phosphorylated Smad2/3 and snail1, and this effect was further enhanced by supplementation with high glucose (Figures 9C,D).

### A high glucose diet exacerbates lung oxidative stress imbalance in the lung of bleomycin-treated mice

**FIGURE 10:**
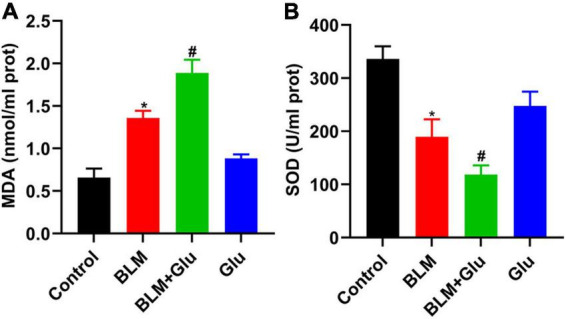
The effect of a high-glucose diet on MDA content and SOD activity in lung tissues. An MDA or SOD kit was used to detect the MDA levels **(A)** and SOD activity **(B)** in lung tissue homogenates. *P < 0.05 vs. control group; ^#^*P* < 0.05 vs. BLM group. The values are means ± SEM (*n* = 8/group). Glu, glucose; BLM, bleomycin.

MDA is the lipid peroxidation product produced by an oxygen-free radical attack on biofilm that indirectly reflects the degree of cell damage. SOD can catalyze the superoxide dismutation reaction, scavenge free radicals in the body, and protect tissues and cells from oxidative damage. MDA content in BLM-treated lung tissue was significantly increased, which was enhanced further by a high glucose diet (Figure 10A). In contrast, SOD activity in lung tissue of mice in the BLM group was significantly reduced, and this was further inhibited when glucose administration (Figure 10B). However, compared to the control group, high glucose diet alone did not increase MDA levels and reduced SOD activity in the lung.

## Discussion

High dietary glucose has been linked to an increased risk of chronic diseases, such as obesity, diabetes, metabolic syndrome, cardiovascular diseases (28–31), and autoimmunity (22). This study investigated the relationship between high glucose and pulmonary fibrosis and demonstrated that high dietary glucose could exacerbate the development of pulmonary fibrosis in the short-term feeding experiment. Further mechanisms revealed that high glucose could promote A549 cell fibrosis due to the TGF-β1 activation *via* high-glucose inducing ROS production and TGF-β signaling *in vitro*. These findings preliminarily uncover a previously unknown risk and mechanism of high dietary glucose in pulmonary fibrosis. Our short-term feeding study found that high dietary glucose worsens BLM-induced IPF by increasing ROS and lung TGF-β1 production. However, the current study cannot determine whether the exacerbation of IPF caused by high glucose intake is mediated *in vivo* directly through the TGF-β signal.

Evidence suggests that ROS plays a “double-edged sword” in tumor cells. ROS, as messenger molecules, are generally produced by cell metabolisms and has a crucial role in modulating cell signals, including normal and cancer cell development, growth, differentiation, and apoptosis. When used as a signaling transducer, ROS can promote cancer cell growth, differentiation, migration, and invasion at adaptable concentrations (32–34). Conversely, high levels of ROS can cause cancer cell apoptosis and eventually death (35), as well as contribute to the TGF-β1-driven persistent pulmonary fibrosis (36, 37). ROS produced by high glucose did not reduce the viability of A549 cells in this study but instead promoted their proliferation, which is consistent with previous reports that showed that high glucose could induce cancer cell growth (38). We further confirmed that high glucose promotes A549 cell growth *via* ROS accumulation because NAC treatment diminished this effect. These findings suggest that high glucose used in this study can produce ROS at a moderate level and ultimately lead to cell growth.

Accumulated evidence suggests that ROS involves in the TGF-β activation (39). TGF-β is typically present in a latent form and can only function when activated (39). Activated TGF-β1 is essential for hyper-glucose-induced fibroblast and epithelial cell hypertrophy (40). Furthermore, high glucose also induces rapid activation of the latent complex of TGF-β by matrix metalloproteinases (40) and ROS (13). *In vitro*, high glucose supplementation induced the EMT of latent TGF-β1-treated-A549 cells by altering their morphology and EMT-related protein expression. Further investigation showed that NAC treatment significantly inhibited high glucose-induced pulmonary fibrosis in latent TGF-β1-treated A549 cells, implying that high-glucose-derived ROS could activate the latent form of TGF-β1 and eventually lead to A549 cell pulmonary fibrosis.

Hyperglycemia environment has been shown to correlate with increased TGF-β activation. High glucose exposure is associated with increased renal interstitial fibrosis (41). Furthermore, hyperglycemia can cause renal insufficiency by inducing oxidative stress, inflammation, and lipid accumulation in the kidneys *via* various signal pathways, which may be critical factors in the pathogenesis of diabetic nephropathy (42). High glucose consumption can promote autoimmune diseases by promoting Th17 cell differentiation, induced by glucose-driven ROS-activated latent TGF-β1 and IL-6 (22). In this study, a high glucose diet exacerbated lung infiltration and fibrosis in BLM-treated mice, accompanied by upregulating ROS and TGF-β1 in the lung *in vivo*. However, high glucose did not change plasma’s TGF-β1 concentration and glucose concentrations (data not shown). Significantly, ROS produced by high glucose did not induce new protein synthesis during the TGF-β activation but rapidly activated the latent TGF-β1 (40). Therefore, our data suggest that high dietary glucose may promote *in vivo* latent TGF-β1 activations of the lung in BLM-treated mice *via* ROS production *in vivo*. However, whether this is true *in vivo* needs to be investigated further.

Mitochondrial DNA is extremely sensitive to oxidative damage, leading to mitochondrial dysfunction and further promoting ROS production, forming an automatic amplification loop of mitochondrial-derived ROS and disrupting mitochondrial homeostasis (43, 44). When mitochondrial dysfunction occurs, mitochondrial superoxide increases DNA damage and stress. High glucose administration significantly increased the amount of mitochondrial ROS and the total amount of ROS in the cells in this and other studies (22). However, based on the differentiation of Th17 cells induced by high glucose, ROS in mitochondria is the key to activating TGF-β, which can induce Th17 cell differentiation, and has nothing to do with the ROS in the cytoplasm (22). The current study found that high glucose promoted the A549 cell proliferation, which may accelerate the EMT. So we hypothesize that the latent TGF-β1 in the high glucose environment mediates pulmonary fibrosis, at least partially *via* ROS derived from mitochondria.

TGF-β will induce lung epithelial cells and fibroblasts differentiated into myofibroblasts, upregulate the mesenchymal marker protein α-SMA expression, accelerate collagen deposition in the lung interstitial, and ultimately cause pulmonary fibrosis after binding to its receptors (TβRI and TβRII) and then activating Smad2/3 phosphorylation (45). Thus, to further determine whether latent TGF-β1 activated by ROS is involved in pulmonary fibrosis *in vitro*, we treated A549 cells in the presence of TGF-β receptor-specific inhibitors SB431542 and found that blocking TβR inhibited collagen I deposition in A549 cells co-treated with latent TGF-β1 and high glucose. Therefore, this verifies that latent TGF-β1 activated by high glucose causes A549 cell differentiation. Unfortunately, this study cannot reveal the effect of high-glucose activated latent TGF-β1 on animal pulmonary fibrosis models.

New studies reveal that changing diets can also alter the progression of pulmonary fibrosis in mice models, except the fact of a high-fat and high-sucrose diet can cause a mouse model of non-alcoholic steatohepatitis with liver fibrosis (46). Mice fed an isoflavone-rich diet, for example, had improved lung function and less lung fibrosis in mice given HCL, indicating the important role of isoflavones content in the rodent diet (47). In BLM-induced mice, a high-fat diet may also delay the development and repair of lung fibrosis (48). In the current work, excessive glucose intake exacerbated the severity of lung fibrosis in mice exposed to BLM. Thus, all of these findings point to the importance of nutrition in the development of lung fibrosis.

This study only accessed the influences of high glucose supplementation on the EMT process in A549 cells and BLM-induced pulmonary fibrosis in mice. Therefore, this effect cannot be determined in the human bronchial epithelial cells or TGF-β1 deficient mice. Another limitation is that mice should be given oxygen-free radical scavengers or inhibitors to explore whether BLM-treated mice supplemented with high glucose will relieve pulmonary fibrosis.

In summary, this study demonstrated that high glucose supplementation increased the production of oxygen free radicals and lung TGF-β1 levels, which could aggravate BLM-induced pulmonary fibrosis in mice. The mechanisms reveal that the effect of high glucose may be due to ROS accumulation and then lead to latent TGF-β1 activation and pulmonary fibrosis. Thus, it may provide more options for better understanding the molecular mechanism of ROS homeostasis defect in treating pulmonary fibrosis.

## Data availability statement

The original contributions presented in this study are included in the article/supplementary material, further inquiries can be directed to the corresponding author.

## Ethics statement

This animal study was reviewed and approved by the Institutional Animal Care and Use Committee of Huaihe Hospital at Henan University.

## Author contributions

JW, JH, YZ, and WN conceived and designed the study. WN, XX, SZ, XW, and HW performed the experiment and analyzed the data. All authors wrote and reviewed the manuscript.

## Abbreviations

BLM: bleomycin
EAE: experimental autoimmune encephalomyelitis
ECM: extracellular matrix
EMT: epithelial–mesenchymal transition
H&E: hematoxylin–eosin
IPF: idiopathic pulmonary fibrosis
Glu: Glucose
L-TGF-β1: latent TGF-β1
MDA: malondialdehyde
NAC: N-Acetyl-L-cysteine
ROS: reactive oxygen species
SOD: superoxide dismutase
α-SMA: α-Smooth muscle actin.
